# Characterization of *Conyza bonariensis* Allelochemicals against Broomrape Weeds

**DOI:** 10.3390/molecules27217421

**Published:** 2022-11-01

**Authors:** Antonio Cala Peralta, Gabriele Soriano, Jesús G. Zorrilla, Marco Masi, Alessio Cimmino, Mónica Fernández-Aparicio

**Affiliations:** 1 Department of Chemical Sciences, University of Naples Federico II, Complesso Universitario Monte S. Angelo, Via Cintia 4, 80126 Naples, Italy; 2 Allelopathy Group, Department of Organic Chemistry, Facultad de Ciencias, Institute of Biomolecules (INBIO), University of Cadiz, C/Avenida República Saharaui, s/n, 11510 Puerto Real, Spain; 3 Department of Plant Breeding, Institute for Sustainable Agriculture (IAS), CSIC, Avenida Menéndez Pidal s/n, 14004 Córdoba, Spain

**Keywords:** *Orobanche*, *Phelipanche*, suicidal germination, radicle growth, parasitic plants, sustainable crop protection

## Abstract

The study of allelopathic activity of plants and the isolation and characterization of the responsible allelochemicals can lead to the development of environment friendly alternative approaches to weed control. *Conyza* species are invasive weeds that use allelopathic activity as part of a successful strategy to outcompete neighboring plants. Broomrape weeds are parasitic plants that use host-induced germination and the formation of a haustorium as strategies to infect host plants. The control of broomrape infection in most affected crops is limited or non-existing. In the current study, we investigated the allelopathic activity of *Conyza bonariensis* organic extracts in suicidal germination and radicle growth of four broomrape species (*Orobanche crenata, Orobanche cumana, Orobanche minor* and *Phelipanche ramosa*). A bioactivity-driven fractionation of *Conyza bonariensis* extracts led to the identification of two germination-inducing molecules and two growth-inhibitory compounds. The germination-inducing metabolites had species-specific activity being hispidulin active on seeds of *O. cumana* and methyl 4-hydroxybenzoate active in *P. ramosa*. The growth-inhibitory metabolites (4*Z*)-lachnophyllum lactone and (4*Z*,8*Z*)-matricaria lactone strongly inhibited the radicle growth of all parasitic weed species studied. Some structure–activity relationships were found as result of the study herein presented.

## 1. Introduction

*Conyza* weeds (Asteraceae) are invasive plants native to America, severely affecting many crops worldwide [1]. *Conyza bonariensis* (L.) Cronq. is present in Spanish crops with noxious effects on their yields [2,3]. *Conyza* species use allelopathy as part of a successful strategy to outcompete neighboring plants [4,5,6,7]. Broomrape weeds (Orobanchaceae) are root-parasitic plants of *Orobanche* and *Phelipanche* genera [8]. Some species of broomrapes are among the most damaging weeds for agricultural production in a large number of crop species. Among them, *Orobanche crenata* infects crops mainly in Fabaceae and Apiaceae. *Orobanche cumana* infects sunflower plants. *Orobanche minor* has a range of host crops in Asteraceae, Apiaceae, Solanaceae and other families. Lastly, *Phelipanche ramosa* infects crops in Solanaceae, Brassicaceae, Cannabaceae, Fabaceae, Apiaceae and Asteraceae [8,9]. Broomrape weeds are obligated parasites that depend on their host crops for nutrient and water supply, and therefore they are obliged to connect with host vasculature shortly after germination [10]. To maximize the likelihood of host connection, their germination is inhibited until perception of metabolites released by roots of potential hosts which ensures the initiation of parasitic weed life cycle in the immediate vicinity of the host [11]. After germination, a tiny radicle emerges through the seed coat that grows towards the host root and then forms a haustorium with functions of host attachment, penetration and vascular connection [12].

In broomrape species, strigolactones are the main class of germination stimulants, being characterized in more than twenty-five structural forms [13,14]. Strigolactones are exuded by crop roots to mediate plant-microbial beneficial interactions [14,15], and collaterally they are used by broomrape weeds to locate the crop root vulnerable to infection. Host-specific broomrapes respond to other chemical classes of germination stimulants. For example, seeds of *O. cumana* germinate in response to sunflower-derived sesquiterpene lactones, i.e., dehydrocostus lactone, costunolide, tomentosin and 8-epixanthatin, or the eudesmanolide anhydrojudaicin [16,17,18]. Germination of *P. ramosa* populations adapted to parasitize Brassicaceae crops responds to glucosinolate-breakdown products, particularly to the isothiocyanates erucin, berteroin, 4-pentenyl isothiocyanate and 2-phenylethyl isothiocyanate [19]. Germination in absence of a host or at a distance longer than 4 mm from the host root is considered suicidal because the seedling exhausts its viability before infection [20,21]. After germination, inhibition of radicle growth impedes the formation of the haustorium on the crop root avoiding crop infection [22]. The mechanisms of host-induced germination and radicle growth towards host root can be targeted by chemical control strategies through the use of natural products either stimulating suicidal germination or inhibiting broomrape radicle growth that minimize infection of crops [22,23].

Two recent studies showed the potential of *Conyza bonariensis* root extracts to inhibit the growth of the parasitic weeds of *Orobanche*, *Phelipanche* [23] and *Cuscuta campestris* (Convolvulaceae) [24]. The *Cuscuta* bioactivity-driven fractionation of *Conyza* extracts allowed the discovery of (4*Z*)-lachnophyllum lactone as the major active component [24]. In the light of this background, this manuscript focuses on the allelochemicals isolated from *C. bonariensis* shoots and reports for the first time the characterization of their activity against four of the most relevant species of broomrape weeds.

## 2. Results and Discussion

### 2.1. Organic Extractions of Conyza Bonariensis Shoots and Evaluation of Inhibitory Activity on Broomrapes

**Figure 1:**
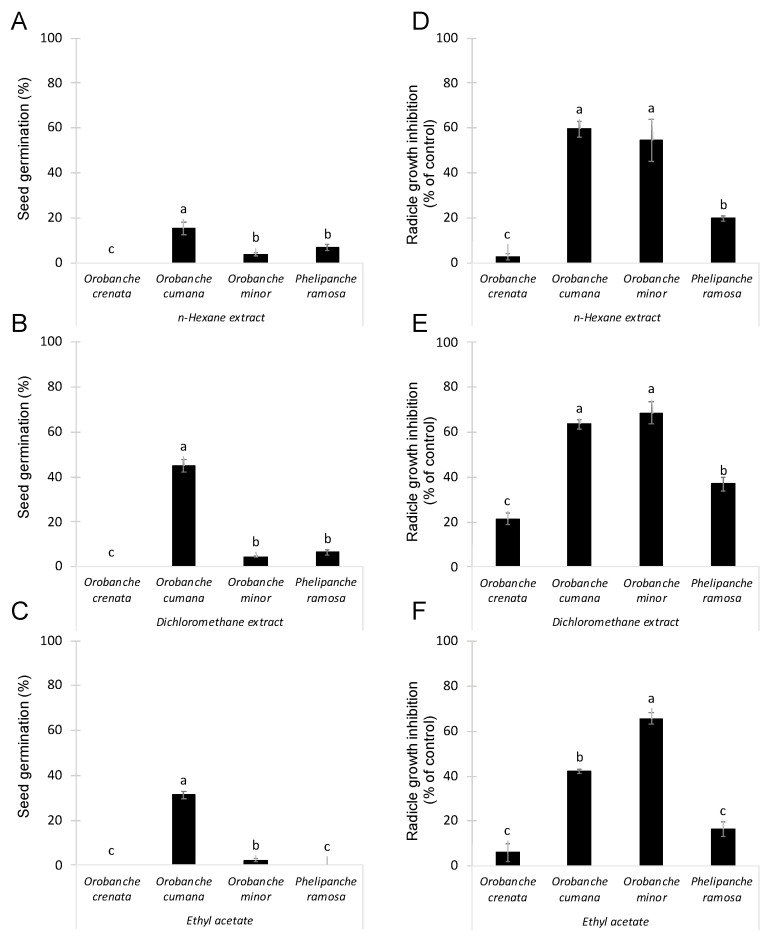
Allelopathic effects on suicidal germination (**A**–**C**) and radicle growth (**D**–**F**) of 4 broomrape species: *Orobanche crenata, Orobanche cumana, Orobanche minor* and *Phelipanche ramosa* induced by extracts prepared from sequential extractions with *n*-hexane (**A**,**D**), dichloromethane (**B**,**E**), and ethyl acetate (**C**,**F**) of *Conyza bonariensis* shoots in each figure, bars with different letters are significantly different according to the Tukey test (*p* < 0.05). Error bars represent the standard error of the mean.

*Conyza bonariensis* lyophilized shoots were extracted by maceration with a hydroalcoholic solution as described in Section 3. Then, three different solvents (namely *n*-hexane, dichloromethane, and ethyl acetate) were used in sequential order to obtain organic extracts containing metabolites with different polarity. The allelopathic activity of these extracts was analysed at 100 μg/mL using two independent bioassays: germination induction and radicle growth bioassays. The germination stimulatory activity of *Conyza* extracts was studied on seeds of four species of broomrapes (*O. crenata*, *O. cumana*, *O. minor* and *P. ramosa*). This germination bioassay was performed diluting each test extract in distilled water. Results are shown in Figure 1 A–C. As expected, null germination was observed when seeds of all broomrape species were treated with negative control (distilled water). Significant effect in germination was observed for *Conyza* extract (ANOVA, *p* < 0.001), for broomrape species (ANOVA, *p* < 0.001) and for the interaction *Conyza* extract x broomrape species (ANOVA, *p* < 0.001). Germination of *O. crenata* seeds were not induced by any of the *Conyza* extracts tested. On the other hand, *Conyza* extracts induced the highest levels of germination on seeds of *O. cumana* (15.3 ± 2.8%, 45.1 ± 2.9% and 31.4 ± 1.6% germination, respectively, induced by *n*-hexane, dichloromethane and ethyl acetate extracts). Low but significant germination was induced by *n*-hexane and dichloromethane extracts in *O. minor* and *P. ramosa* seeds (Figure 1A,B) and by ethyl acetate extracts in *O. minor* seeds (Figure 1C).

The growth inhibitory activity of *Conyza* extracts was studied on radicles of the four broomrapes species (*O. crenata*, *O. cumana*, *O. minor* and *P. ramosa*). As germination of broomrape seeds is inhibited until detection of germination stimulants, the radicle growth bioassay was performed mixing each test extract with the germination stimulant GR24. Results are shown in Figure 1D–F. Significant effect in radicle growth inhibition was observed for *Conyza* extract (ANOVA, *p* < 0.001), for broomrape species (ANOVA, *p* < 0.001) and for the interaction *Conyza* extract x broomrape species (ANOVA, *p* = 0.023). All extracts induced the highest growth inhibitory activity on radicles of *O. cumana* (59.4 ± 3.3%, 63.7 ± 2.1%, and 42.0 ± 0.9% of inhibition, respectively, observed with treatments of *n*-hexane, dichloromethane and ethyl acetate extracts) and *O. minor* (54.5 ± 9.3%, 68.7 ± 4.9% and 65.6 ± 2.7% of inhibition, respectively, observed with treatments of *n*-hexane, dichloromethane and ethyl acetate extracts). All extracts induced moderate levels of inhibition in *P. ramosa* radicles and low or negligible in *O. crenata* radicles (Figure 1D–F).

### 2.2. Isolation of Pure Metabolites (**1**–**7**) from the Organic Extracts and their Chemical Identification

**Figure 2:**
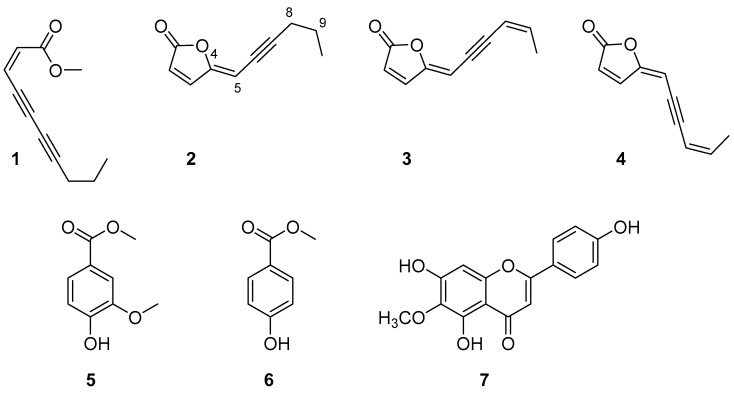
Compounds isolated from *Conyza bonariensis* shoot extracts.

Once the activity of the organic extracts was confirmed, they were further purified as described in Section 3. Seven pure metabolites (**1**–**7**, Figure 2) were isolated and identified as (4*Z*)-lachnophyllum methyl ester (**1**, 26.0 mg), (4*Z*)-lachnophyllum lactone (**2**, 45.9 mg), (4*Z*,8*Z*)-matricaria lactone (**3**, 9.3 mg), (4*E*,8*Z*)-matricaria lactone (**4**, 3.8 mg), methyl 4-hydroxy-3-methoxybenzoate (**5**, 9.8 mg), methyl 4-hydroxybenzoate (**6**, 13.8 mg) and hispidulin (**7**, 9.6 mg) (Figure 2). Specifically, the purification of the *n*-hexane extract provided compounds **1**–**4**, the CH_2_Cl_2_ extract provided compounds **2** and **5**–**7**, and the EtOAc extract provided compounds **5**–**7**.

The structures of the isolated compounds were confirmed by NMR spectroscopy and MS and by comparison with the data reported in the literature (see Section 3.3). The ^1^H NMR spectrum and the molecular ion peak of compound **1** indicated the obtaining of (4*Z*)-lachnophyllum methyl ester, a compound previously found in *C. bonariensis* [25], whose structure would correspond with that of the opening of a furanone lactone. Instead, compounds **2**–**4** were lactones constituted by a 2-furanone ring bonded to an unsaturated chain. The signals assigned to the hydrogen atoms of the chain in the ^1^H NMR spectra, and the close molecular ion peak values obtained for these three compounds (*m/z*: 161–163), indicated that compounds **2**–**4** differed from each other in the level of unsaturation or in the geometry of the double bonds. Thus, compound **2** was identified as (4*Z*)-lachnophyllum lactone [24], whereas compounds **3** and **4** were analogues of compound **2** containing a double bond in positions C_8_ = C_9_, whose structure corresponds with that of matricaria lactone. By comparing the ^1^H NMR spectra of compounds **3** and **4** with the data available in the literature for matricaria lactone, the main difference observed was that H-5 appeared 0.43 ppm higher in compound **3**. Thus, it was concluded that compounds **3** and **4** were geometric isomers at their C_4_ = C_5_ double bonds, being identified as (4*Z*,8*Z*)-matricaria lactone (**3**) and (4*E*,8*Z*)-matricaria lactone (**4**) [26,27].

The ^1^H NMR spectra of compounds **5** and **6** indicated that both compounds were benzene-derived aromatic compounds. Attending also at their molecular ion peak values, it was confirmed that both compounds contain a hydroxyl and a methyl ester group in para positions and that compound **5** shows an additional *ortho*-methoxy group. They were identified as methyl 4-hydroxy-3-methoxybenzoate (**5**) [28] and methyl 4-hydroxybenzoate (**6**) [29].

The experimental NMR spectra and molecular ion peak of compound **7** were in agreement with that of a flavonoid containing three hydroxyl groups and one methoxy group as substituents. It was identified as hispidulin by comparison of its data with that already reported for this flavonoid [30,31]. It is worth noting that hispidulin is a flavonoid with anti-inflammatory and antioxidant properties [32]. It has potential use as an anticancer drug [33], also being found with activity as a quorum sensing inhibitor with application in the control of infections caused by *Pseudomonas aeruginosa* [34] and with activity on phytotoxic assays on the root and seedling growth and on the seed germination of crop species of radish, cucumber and onion [35].

### 2.3. Bioactivity of the Isolated Compounds (**1**–**7**) on Broomrapes

Allelopathic effects of compounds **1**–**7** were analysed on four broomrapes species (*O. crenata*, *O. cumana*, *O. minor* and *P. ramosa*) at 1 and 0.1 mM using two independent bioassays: germination induction and radicle growth inhibition bioassays. The germination bioassay was performed diluting each molecule in distilled water. As expected, null germination was observed when seeds of all broomrape species were treated with negative control (distilled water). High germination activity was observed by the positive control, the synthetic strigolactone GR24 (71.1 ± 1.4%, 63.5 ± 1.2%, 80.5 ± 2.0% and 74.9 ± 1.3% of germination of *O. crenata*, *O. cumana*, *O. minor* and *P. ramosa,* respectively). Among the isolated compounds, methyl 4-hydroxybenzoate (**6**) and hispidulin (**7**) showed significant stimulatory activity. Specifically, compound **6** was active on *P. ramosa* when applied at 1 mM (58.1 ± 3.0% of seed germination) and at 0.1 mM (26.7 ± 1.9% of seed germination), though null germination was observed for the rest of broomrape species studied (data not shown). The absence of methyl 4-hydroxy-3-methoxybenzoate (**5**) activity showed that the *p*-methoxy group has a direct influence on the loss of the activity observed for compound **5** in comparison with compound **6**. Similar loss of activity in flavonoid-induced *Gigaspora rosea* germination was observed by Scervino et al. [36]. Compound **7** showed significant stimulatory activity on *O. cumana* when applied at 1mM (31.2 ± 2.7% of seed germination) and at 0.1 mM (5.3 ± 14% of seed germination), whereas null germination was observed in the rest of broomrape species studied. Suicidal germination of root parasitic weeds has been previously reported to be induced by the isoflavone uncinanone B isolated from *Desmodium uncinatum* [37].

**Figure 3:**
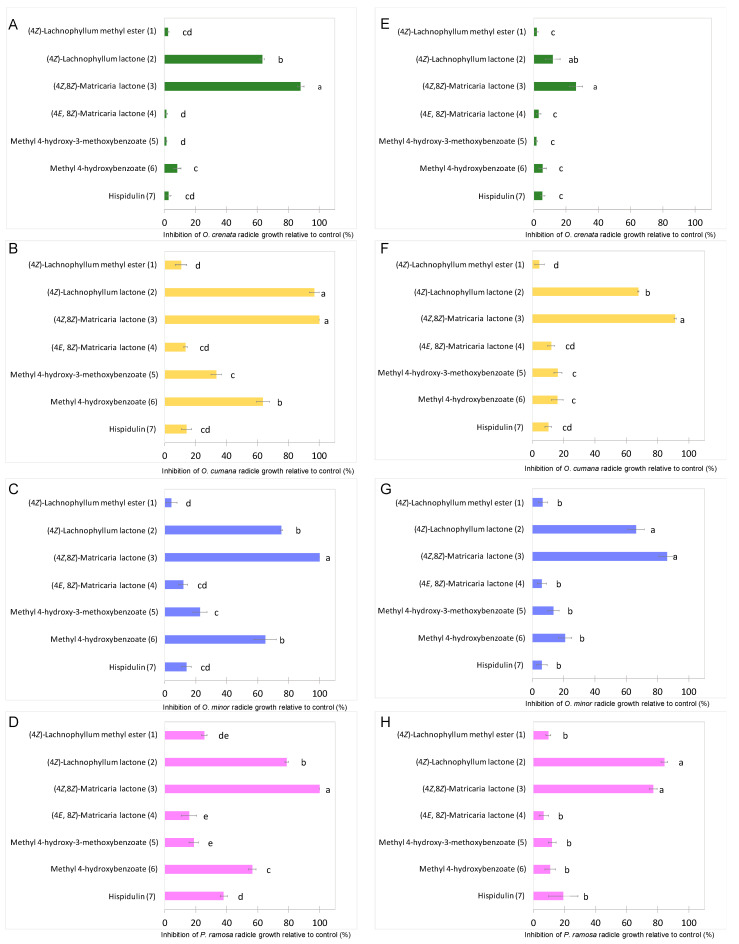
Growth inhibition induced by (4*Z*)-lachnophyllum methyl ester (**1**); (4*Z*)-lachnophyllum lactone (**2**); (4*Z*,8*Z*)-matricaria lactone (**3**), (4*E*,8*Z*)-matricaria lactone (**4**); methyl 4-hydroxy-3-methoxybenzoate (**5**); methyl 4-hydroxybenzoate (**6**); hispidulin (**7**); applied at 1mM (**A**–**D**); and 0.1mM (**E**–**H**); in radicles of *O. crenata* (**A**,**E**); *O. cumana* (**B**,**F**); *O. minor* (**C**,**G**); and *P. ramosa* (**D**,**H**); bars with different letters are significantly different according to the Tukey test (*p* < 0.05). Error bars represent the standard error of the mean.

**Figure 4:**
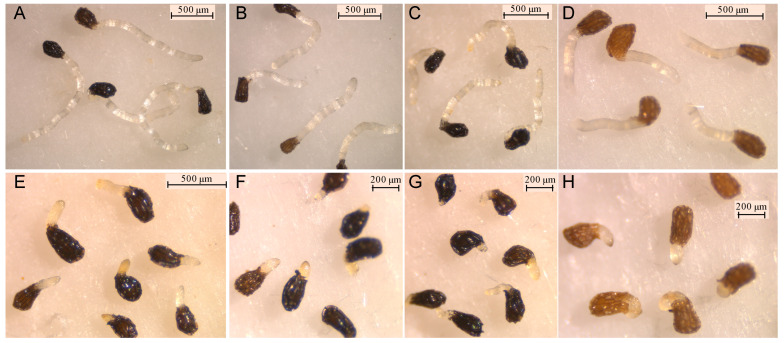
Treatments with control (**A**–**D**) and compound (4*Z*,8*Z*)-matricaria lactone (**3**) applied at 0.1mM (**E**–**H**) on radicles of *O. crenata* (**A**,**E**); radicles of *O. cumana* (**B**,**F**); radicles of *O. minor* (**C**,**G**); and radicles of *P. ramosa* (**D**,**H**).

The growth inhibitory activity of compounds **1**–**7** was studied on radicles of *O. crenata*, *O. cumana*, *O. minor* and *P. ramosa*. As germination of broomrape seeds is naturally inhibited until detection of germination stimulants, the radicle growth bioassay was performed mixing each test compound with the germination stimulant GR24. Results are shown on Figure 3. Significant effect in radicle growth inhibition was observed for the type of compound (ANOVA, *p* < 0.001), for broomrape species (ANOVA, *p* < 0.001), for compound concentration (ANOVA, *p* < 0.001) and for the interaction compound x broomrape species (ANOVA, *p* < 0.001) or the interaction compound x concentration (ANOVA, *p* < 0.001). The (4*Z*)-lachnophyllum lactone (**2**) and (4*Z*,8*Z*)-matricaria lactone (**3**) were the most active compounds both at concentrations of 1 mM (Figure 3A–D) and 0.1 mM (Figure 3E–H). In all broomrape species tested, compound **3** showed the strongest inhibition activity on radicles (Figure 4), with inhibition values over 80% in most of the cases. At 1mM, inhibition values close to 100% were observed for *O. cumana*, *O. minor* and *P. ramosa*. The activity showed by compound **2** is also worth highlighting, with inhibition values over 70% in most of the cases. These results, together with that recently reported against *C. campestris* [24], support the potential of (4*Z*)-lachnophyllum lactone (**2**) as a promising allelochemical for parasitic weed control.

**Figure 5:**
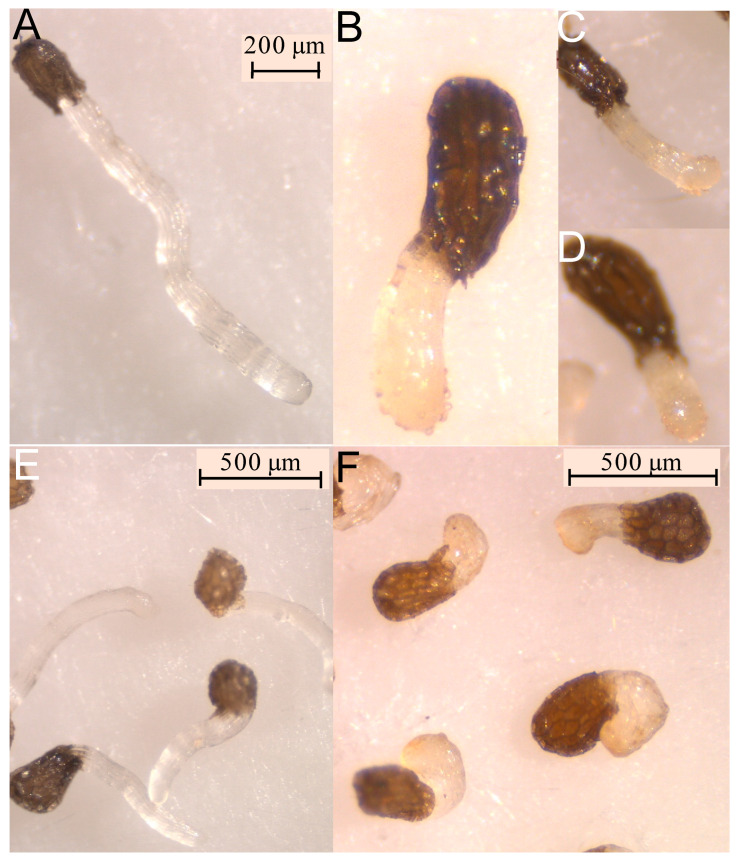
Radicles of *O. cumana* (**A**–**D**) and P*. ramosa* (**E**,**F**) treated with control (**A**,**E**) and compound methyl 4-hydroxybenzoate (**6**) at 1 mM.

By comparing the activity of compounds **2** and **3** with that of the other tested compounds (Figure 3), a remarkable improvement, especially when tested at the lowest concentration (0.1 mM), can be observed. Methyl 4-hydroxybenzoate (**6**) also showed to be moderately active at reducing radicle growth inhibition at 1 mM for *O. cumana*, *O. minor* and *P. ramosa*, with inhibition values around 60% (Figure 3B–D, Figure 5).

At this concentration, formation of papillae at the tip of the treated radicles was also observed in *O. cumana* (Figure 5B–D), while a smooth radicle tip without papillae was observed in *O. cumana* radicles treated with control (Figure 5A). The activity of radicle growth inhibition and papillae formation of compound **6** was lost at a concentration of 0.1 mM. Low or negligible activity was observed for the rest of the tested compounds at all concentrations tested (**1**, **4**, **5** and **7**).

From the structural point of view, it could be concluded that the high inhibition activity levels showed by the lactones (4*Z*)-lachnophyllum lactone (**2**) and (4*Z*,8*Z*)-matricaria lactone (**3**) decrease to a high extent when the lactonic ring is opened (compound **1**). This conclusion is in agreement with the results reported in a previous study for the same compounds in inhibition growth bioassays on monocot and dicot species [26]. Comparing the activity levels of compounds **3** (4*Z*) and **4** (4*E*), a strong effect of the geometry of the C_4_ = C_5_ double bond can also be attributed to the loss of activity of compound **4**. These conclusions obtained for compounds **1**–**4** can be completed by a comparison with their respective lipophilicity values, calculated by the CLog*P* algorithm, to provide some glimpse of their different behavior. Respectively, these CLog*P* values are 2.71 (**1**), 2.46 (**2**) and 2.00 (**3** and **4**). It can be observed that the lower activity of compound **1** could be related to its higher lipophilicity, possessing a different solubility than compounds **2**–**4**. When comparing the geometric isomer compounds **3** and **4**, similar CLog*P* values are calculated. This result indicates that the great difference of activity between both compounds would be related to a different reactivity and not to different levels of solubility.

Finally, the results obtained for the aromatic compounds **5** and **6** allow us to conclude that the presence of the *p*-methoxy group is related to a decrease of the inhibition activity. This structural relationship, as previously mentioned, was also observed in the germination induction bioassay.

## 3. Materials and Methods

### 3.1. General Experimental Procedures

^1^H and ^13^C NMR spectra were recorded at 500/125 MHz on a Bruker 500 AVANCE NEO spectrometer (Karlsruhe, Germany) or at 400/100 MHz on a Bruker 400 Anova Advance. The spectra were recorded using CDCl_3_ or (CD_3_)_2_CO, and the same solvents were used as internal standards. Electrospray ionization mass spectra (ESIMS) were performed using the LC/MS TOF system AGILENT 6230B (Agilent Technologies, Milan, Italy), HPLC 1260 Infinity. Column chromatography (CC) was performed using silica gel (Kieselgel 60, 0.063–0.200 mm, Merck, Darmstadt, Germany). Thin-layer chromatography (TLC) was performed on analytical and preparative silica gel plates (Kieselgel 60, F_254_, 0.25 and 0.5 mm, respectively, Merck, Darmstadt, Germany). The spots were visualized via exposure to UV light (254 nm) and/or iodine vapours and/or by spraying first with 10% H_2_SO_4_ in MeOH and then with 5% phosphomolybdic acid in EtOH, followed by heating at 110 °C for 10 min. Sigma-Aldrich Co. (St. Louis, MO, USA) supplied all the reagents and the solvents.

### 3.2. Plant Material

Specimens of *Conyza bonariensis* were harvested at the phenological stage of emergence of the inflorescence in spring of 2022 in Cordoba, southern Spain (coordinates 37.856 N, 4.806 W, datum WGS84). After harvesting, *C. bonariensis* shoots were immediately carried to the laboratory and frozen with liquid nitrogen, stored at −80 °C, subsequently lyophilized and the dry material stored until use at 4 °C in the dark. Broomrape seeds were collected from mature plants of *Orobanche crenata* infecting pea plants in Spain*, Orobanche cumana* infecting sunflower plants in Spain, *Orobanche minor* infecting red clover plants in France and *Phelipanche ramosa* infecting tobacco plants in France. Dry parasitic seeds were separated from capsules using a sieve of 0.6 mm mesh size and then stored dry at room temperature in the dark until use for this work.

### 3.3. Isolation and Identification of Metabolites from Conyza bonariensis Shoots

A total of 240 g of *Conyza bonariensis* shoots were extracted, following a previously reported protocol often used for the extraction of plant material [38]. In particular, the shoots were ground and extracted by 1.7 L H_2_O/MeOH (1/1, *v/v*), under stirred conditions at room temperature for 24 h. The hydroalcoholic suspensions were centrifuged at 7000 rpm and extracted with *n*-hexane (3 × 800 mL), CHCl_3_ (3 × 800 mL) after removing methanol under reduced pressure, with EtOAc (3 × 500 mL). Each extract was dried over anhydrous Na_2_SO_4_, then filtered, and the solvent was evaporated under reduced pressure. The extraction was carried twice, and each organic extract was combined, yielding 169.1 mg (*n*-hexane), 276.1 mg (CHCl_3_) and 295.9 mg (EtOAc).

The *n*-hexane organic extract was purified by CC on Si-gel, eluted with CHCl_3_/*i*-propanol (9/1, *v/v*), yielding seven homogeneous fractions (F1-F7). The residue of F2 (41.7 mg) was purified by TLC eluted with *n*-hexane/EtOAc (9/1, *v/v*), yielding five groups of homogeneous fractions (F1.1-F1.5). The residue of F1.4 (47.1 mg) was purified by TLC eluted with *n*-hexane/EtOAc (95/5, *v/v*), yielding a pure compound identified as (4*Z*)-lachnophyllum methyl ester (**1**, 10.1 mg). The residue of F3 (93.0 mg) was purified by TLC eluted with *n*-hexane/EtOAc (4/1, *v/v*), yielding four groups of homogeneous fractions (F3.1-F3.4). F3.1 yielded a further amount of compound **1** (15.9 mg, for a total amount of 26.0 mg). The residue of F3.2 was identified as (4*Z*)-lachnophyllum lactone (**2,** 32.6 mg), the residue of F3.3 was identified as (4*E*,8*Z*)-matricaria lactone (**4**, 3.8 mg), while F3.4 was identified as (4*Z*,8*Z*)-matricaria lactone (**3**, 9.3 mg).

The CH_2_Cl_2_ organic extract was purified by CC on Si-gel, eluted with CHCl_3_/*i*-propanol (95/5, *v/v*), yielding nine homogeneous fractions (F1-F9). The residue of F1 (32.2 mg) was further purified by TLC eluted *n*-hexane/EtOAc (4/1, *v/v*), yielding a further amount of (4*Z*)-lachnophyllum lactone (**2,** 13.3 mg, for a total amount of 45.9 mg). The residue of F4 (42.6 mg) was purified by TLC eluted with CHCl_3_/*i*-propanol (9/1, *v/v*), yielding two pure compounds identified as methyl 4-hydroxy-3-methoxybenzoate (methyl vanillate, **5**, 4.8 mg) and as methyl 4-hydroxybenzoate (**6**, 6.4 mg). The residue of F5 (34.6 mg) was purified by TLC eluted with CHCl_3_/*i*-propanol (98/2, *v/v*), yielding a pure compound identified as hispidulin (**7**, 6.2 mg).

The EtOAc organic extract was purified by CC on Si-gel, eluted with CHCl_3_/*i*-propanol (95/5, *v/v*), yielding six homogeneous fractions (F1-F6). The residue of F1 (37.6 mg) was further purified by TLC eluted with CHCl_3_/*i*-propanol (98/2, *v/v*), yielding a further amount of methyl 4-hydroxy-3-methoxybenzoate (**5**, 5.0 mg, for a total amount of 9.8 mg) and of methyl 4-hydroxybenzoate (**6**, 7.4 mg, for a total amount of 13.8 mg). The residue of F2 (12.6 mg) was purified by TLC eluted with CHCl_3_/*i*-propanol (98/2, *v/v*), yielding further amount of hispidulin (**7**, 3.4 mg, for a total amount of 9.6 mg).

(4*Z*)-Lachnophyllum methyl ester (**1**): ^1^H NMR spectrum (Supplementary materials Figure S1) was in agreement with data previously reported [25]. ESI MS (+) *m/z*: 177 [M+H]^+^ (Figure S2).

(4*Z*)-Lachnophyllum lactone (**2**): ^1^H NMR spectrum (Figure S3) was in agreement with data previously reported [24]. ESI MS (+) *m/z*: 163 [M+H]^+^ (Figure S4).

(4*Z*,8*Z*)-Matricaria lactone (**3**): ^1^H NMR spectrum (Figure S5) was in agreement with data previously reported [26]. ESI MS (+) *m/z*: 161 [M+H]^+^ (Figure S6).

(4*E*,8*Z*)-Matricaria lactone (**4**): ^1^H NMR spectrum (Figure S7) were in agreement with data previously reported [27]. ESI MS (+) *m/z*: 161 [M+H]^+^ (Figure S8).

Methyl 4-hydroxy-3-methoxybenzoate (**5**): ^1^H NMR spectrum (Figure S9) was in agreement with data previously reported [28]. ESI MS (+) *m/z*: 183 [M+H]^+^ (Figure S10).

Methyl 4-hydroxybenzoate (**6**): ^1^H NMR spectrum (Figure S11) was in agreement with data previously reported [29]. ESI MS (+) *m/z*: 153 [M+H]^+^ (Figure S12).

Hispidulin (**7**): ^1^H NMR and ^13^C NMR spectra (Figures S13 and S14) were in agreement with data previously reported [30,31]. ESI MS (+) *m/z*: 301 [M+H]^+^ (Figure S15).

### 3.4. Bioactivity on Parasitic Weed Seeds

Allelopathic effects of each *Conyza bonariensis* extract and isolated compounds were tested on broomrape seeds in two independent bioassays conducted according to previous protocols [39]. First, seeds of four broomrape species, *Orobanche crenata*, *Orobanche cumana*, *Orobanche minor* and *Phelipanche ramosa,* were surface-sterilized by immersion in 0.5% (*w*/*v*) NaOCl and 0.02% (*v/v*) Tween 20, for 5 min, rinsed with sterile distilled water, and dried in a laminar airflow cabinet. Previous to germination induction, broomrape seeds require to be conditioned using a warm stratification. Approximately 100 seeds of each broomrape species were individually placed on each of a total of 105 glass fiber filter paper discs (GFFP) (Whatman International Ltd., Maidstone, UK) of 9 mm-diameter, each one moistened with 50 μL of sterile distilled water, and placed in Petri dishes sealed with parafilm in incubators at 23 °C for 10 days.

For the assay of suicidal germination, for each broomrape species 42 GFFP discs containing conditioned seeds were placed onto a sterile sheet of filter paper to remove the conditioning water and transferred dry to new 9 cm sterile Petri dishes. Stock solutions of each *Conyza* extract and purified metabolite were dissolved in dimethyl sulfoxide and then individually diluted in sterile distilled water up to an equivalent concentration of 100 μg/mL in the case of the extracts and 1 mM in the case of purified metabolites (**1–7**). Triplicate aliquots of treatments containing only sterile distilled water supplemented with 2% dimethyl sulfoxide were used as a negative control, and the synthetic germination stimulant GR24 supplemented with 2% dimethyl sulfoxide was used as a positive control.

For the assay of radicle growth inhibition, for each broomrape species 63 GFFP discs containing the conditioned seeds were placed onto a sterile sheet of filter paper to remove the conditioning water and transferred dry to new 9 cm sterile Petri dishes. Broomrape seeds require the induction of germination with a germination stimulant and therefore for radicle growth bioassays, the test compound is applied to broomrape seeds mixed with the synthetic germination stimulant GR24. Stock solutions of each extract and purified metabolite were dissolved in dimethyl sulfoxide and then diluted using an aqueous solution of GR24 up to a concentration of 100 μg/mL in the case of the *Conyza* extracts and at concentrations of 1 and 0.1 mM in the case of purified metabolites **1–7**. For each assay, triplicate aliquots of each sample were applied to GFFP discs containing conditioned seeds of each broomrape species. Triplicate aliquots of treatments only containing GR24 supplemented with 2% dimethyl sulfoxide were used as a control.

Treated seeds were incubated in the dark at 23 °C for 7 days, and the percent of germination and radicle growth was determined for each GFFP disc, using a stereoscopic microscope (Leica S9i, Leica Microsystems GmbH, Wetzlar, Germany). For suicidal germination assays, the activity of each extract and purified metabolite was determined by counting the percent of germinated seeds for each GFFP disk. For the characteristic of radicle growth inhibition activity of each extract and purified metabolite, the value used was the average of 10 randomly selected radicles per GFFP disc [40]. The percentage of radicle growth inhibition of each treatment was then calculated relative to the average radicle growth of control treatment.

### 3.5. CLogP

CLog*P* were calculated using ChemOffice v20.1 (PerkinElmer, Waltham, MA, USA) by means of the appropriate tool in ChemDraw Professional [41].

### 3.6. Data Analyses

All bioassays were performed using a completely randomized design. Percentage data in *Orobanche* assays were approximated to normal frequency distribution by means of angular transformation and subjected to analysis of variance (ANOVA) using SPSS software for Windows (SPSS Inc., Chicago, IL, USA). The significance of mean differences among treatments was evaluated by the Tukey test. The null hypothesis was rejected at the level of 0.05.

## 4. Conclusions

This study shows the potential of some of the metabolites produced by *C. bonariensis* to be studied in depth as allelochemicals for the control of parasitic weeds of the broomrape species. Methyl 4-hydroxybenzoate (**6**) and hispidulin (**7**) showed to be active for inducing the germination of *P. ramosa* or *O. cumana*, so both compounds would be of interest to be used in the suicidal germination strategy. In order to explore the possibilities of the isolated compounds as growth inhibitors, it was found that (4*Z*)-lachnophyllum lactone (**2**) and (4*Z*,8*Z*)-matricaria lactone (**3**) strongly inhibited the radicle growth of all parasitic weed species studied. Since compound 2 was isolated in a higher yield, this allelochemical represents a special promising and accessible tool for the management of broomrape pests. From the structural point of view, the change of geometry to 4*E* and the opening of the lactone ring of compounds **2** and **3** have been found detrimental for their growth inhibitory activity.

## Data Availability

Not applicable.

## Supplementary Materials

The following are available online at https://www.mdpi.com/article/10.3390/molecules27217421/s1; Figure S1: ^1^H-NMR spectrum of **1** (CDCl_3_, 500 MHz); Figure S2: ESI MS spectrum of **1**; Figure S3: ^1^H-NMR spectrum of **2** (CDCl_3_, 500 MHz); Figure S4: ESI MS spectrum of **2**; Figure S5: ^1^H-NMR spectrum of **3** (CDCl_3_, 500 MHz); Figure S6: ESI MS spectrum of **3**; Figure S7: ^1^H-NMR spectrum of **4** (CDCl_3_, 500 MHz); Figure S8: ESI MS spectrum of **4**; Figure S9: ^1^H-NMR spectrum of **5** (CDCl_3_, 500 MHz); Figure S10: ESI MS spectrum of **5**; Figure S11: ^1^H-NMR spectrum of **6** (CDCl_3_, 500 MHz); Figure S12: ESI MS spectrum of **6**; Figure S13: ^1^H-NMR spectrum of **7** (CDCl_3_, 500 MHz); Figure S14: ^13^C-NMR spectrum of **7** (CDCl_3_, 125 MHz); Figure S15: ESI MS spectrum of **7**.

## Sample Availability

Samples of the compounds are available from the authors.
