# Supplementary material for: Characterization of Conyza bonariensis Allelochemicals against Broomrape Weeds
Source: Molecules. 2022 Nov 1;27(21):7421. doi: 10.3390/molecules27217421 (PMC9654463; doi:10.3390/molecules27217421)
Supplement: Supplementary file 1 [file molecules-27-07421-s001.zip › molecules-1979307-supplementary.pdf]

Supporting Information

Characterization of *Conyza bonariensis* allelochemicals against broomrape weeds

Antonio Cala Peralta[1,2], Gabriele Soriano[2], Jesús G. Zorrilla[1,2], Marco Masi[2,*], Alessio Cimmino[2] , Mónica Fernández-Aparicio[3,*]

[1] Department of Chemical Sciences, University of Naples Federico II, Complesso Universitario Monte S. Angelo, Via Cintia 4, 80126 Naples, Italy; antonio.cala@uca.es (A.C.P.); gabriele.soriano@unina.it (G.S.); jesus.garcia@uca.es (J.G.Z.); alessio.cimmino@unina.it (A.C.)
[2] Allelopathy Group, Department of Organic Chemistry, Facultad de Ciencias, Institute of Biomolecules (INBIO), University of Cadiz, C/Avenida República Saharaui, s/n, 11510 Puerto Real, Spain
[3] Department of Plant Breeding, Institute for Sustainable Agriculture (IAS), CSIC, Avenida Menéndez Pidal s/n, 14004 Córdoba, Spain
* Correspondence: marco.masi@unina.it (M.M.); monica.fernandez@ias.csic.es (M.F.-A.)

Supporting Information list

Figure S1. ^{1}H-NMR spectrum of (4*Z*)-lachnophyllum methyl ester (**1**) recorded in $CDCl_3$ at 500 MHz.

Figure S2. ESI MS spectrum of (4*Z*)-lachnophyllum methyl ester (**1**) recorded in positive modality.

Figure S3. ^{1}H-NMR spectrum of (4*Z*)-lachnophyllum lactone (**2**) recorded in $CDCl_3$ at 500 MHz.

Figure S4. ESI MS spectrum of (4*Z*)-lachnophyllum lactone (**2**) recorded in positive modality.

Figure S5. ^{1}H-NMR spectrum of (4*Z*,8*Z*)-matricaria lactone (**3**) recorded in $CDCl_3$ at 500 MHz.

Figure S6. ESI MS spectrum of (4*Z*,8*Z*)-matricaria lactone (**3**) recorded in positive modality.

Figure S7. ^{1}H-NMR spectrum of (4*E*,8*Z*)-matricaria lactone (**4**) recorded in $CDCl_3$ at 500 MHz.

Figure S8. ESI MS spectrum of (4*E*,8*Z*)-matricaria lactone (**4**) recorded in positive modality.

Figure S9. ^{1}H-NMR spectrum of methyl 4-hydroxy-3-methoxybenzoate (**5**) recorded in $CDCl_3$ at 500 MHz.

Figure S10. ESI MS spectrum of methyl 4-hydroxy-3-methoxybenzoate (**5**) recorded in positive modality.

Figure S11. ^{1}H-NMR spectrum of methyl 4-hydroxybenzoate (**6**) recorded in $CDCl_3$ at 500 MHz.

Figure S12. ESI MS spectrum of methyl 4-hydroxybenzoate (**6**) recorded in positive modality.

Figure S13. ^{1}H-NMR spectrum of hispidulin (**7**) recorded in $CDCl_3$ at 500 MHz.

Figure S14. ^{13}C-NMR spectrum of hispidulin (**7**) recorded in $(CD_3)_2CO$ at 125 MHz.

Figure S15. ESI MS spectrum of hispidulin (**7**) recorded in positive modality.

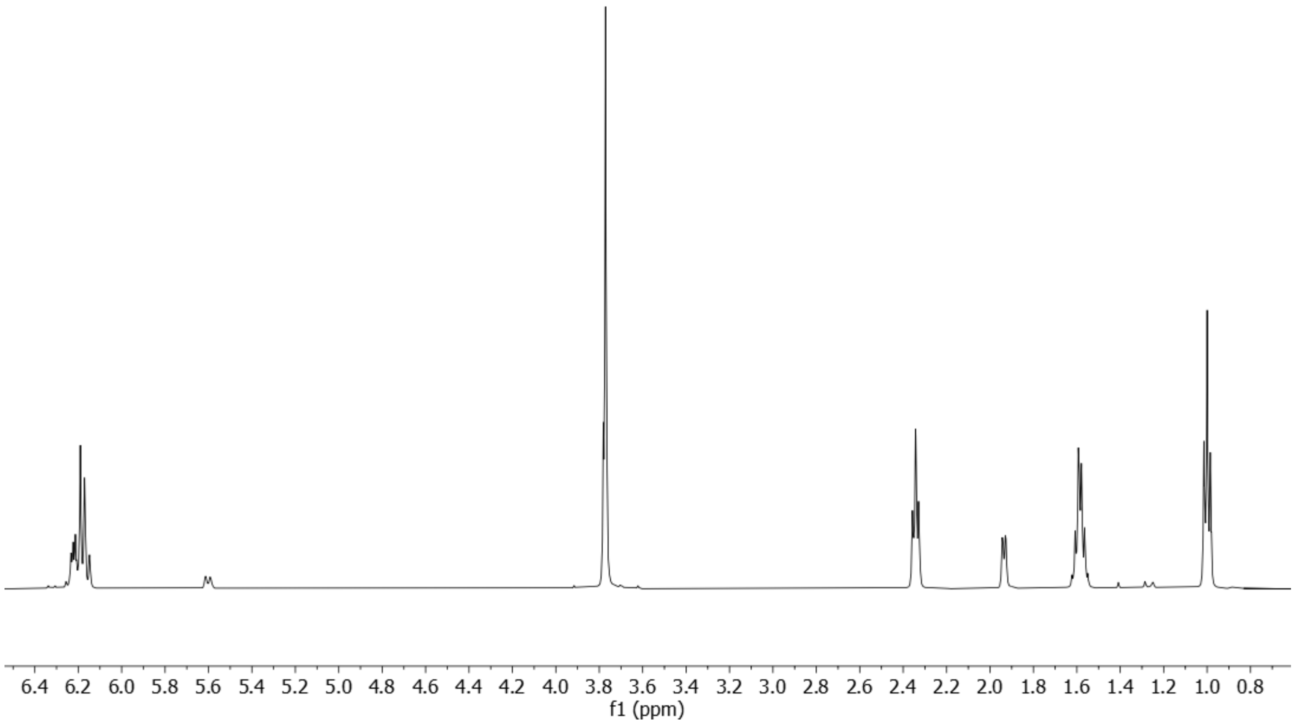

Figure S1. ^{1}H-NMR spectrum of (4*Z*)-lachnophyllum methyl ester (**1**) recorded in $CDCl_3$ at 500 MHz.

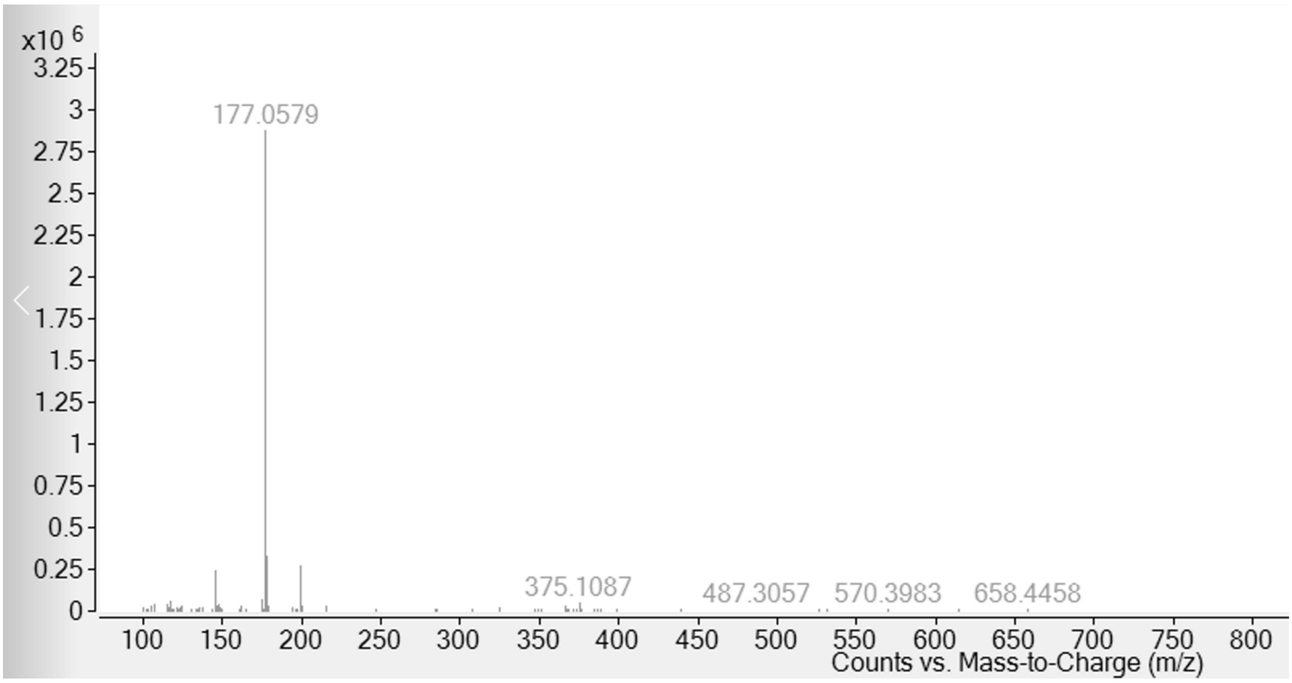

Figure S2. ESI MS spectrum of (4*Z*)-lachnophyllum methyl ester (**1**) recorded in positive modality.

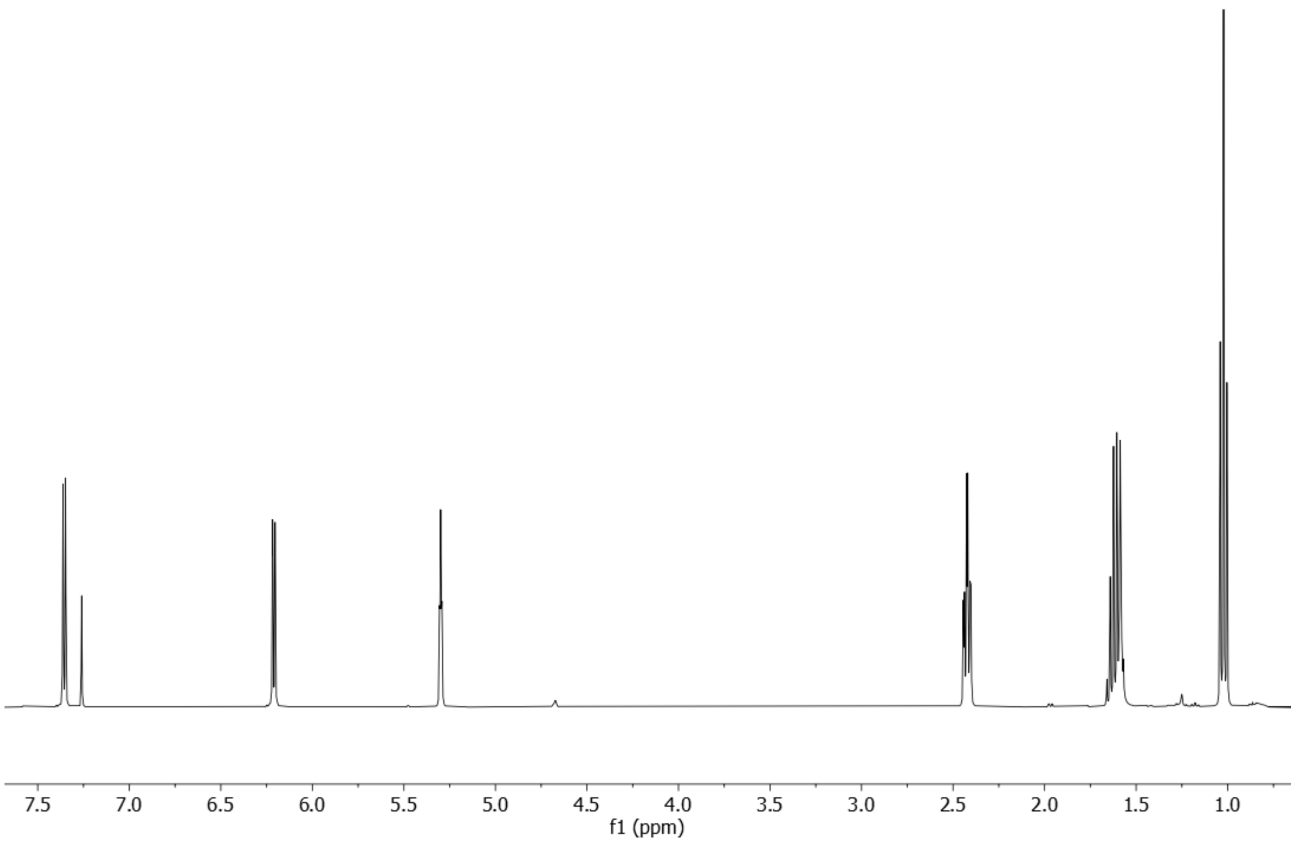

Figure S3. ^{1}H-NMR spectrum of (4*Z*)-lachnophyllum lactone (**2**) recorded in $CDCl_3$ at 500 MHz.

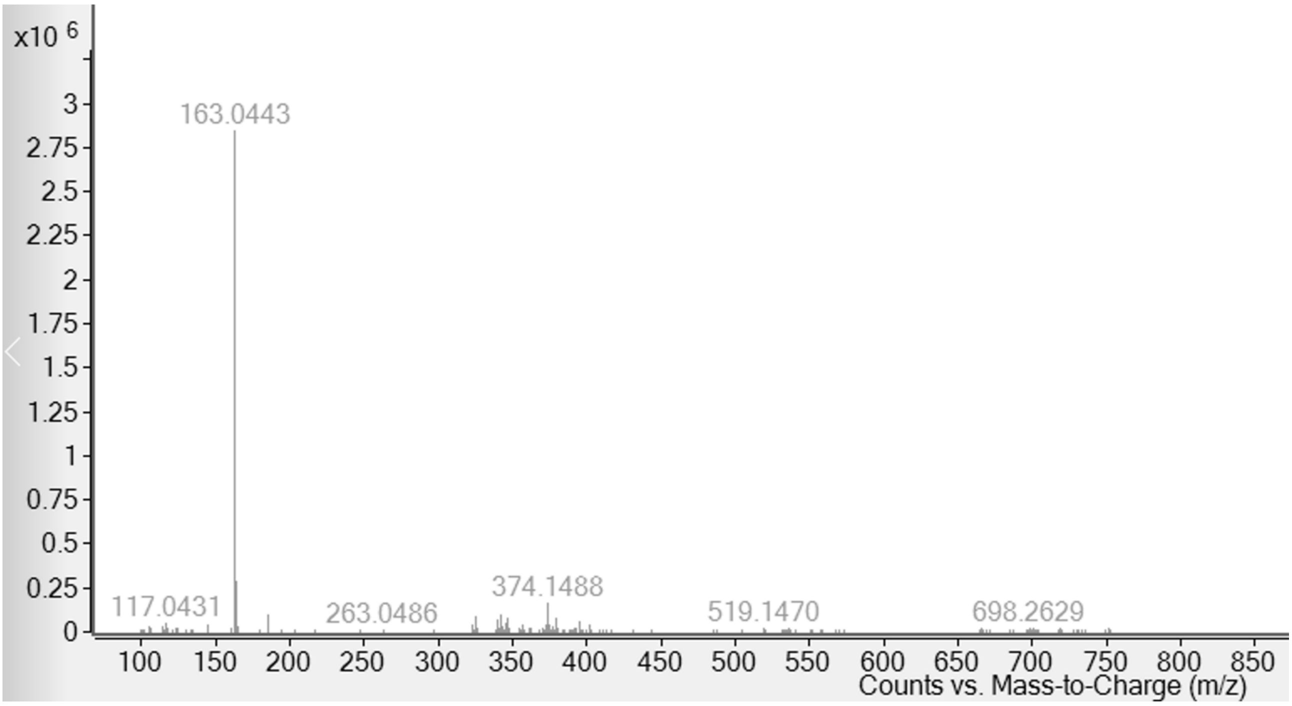

Figure S4. ESI MS spectrum of (4*Z*)-lachnophyllum lactone (**2**) recorded in positive modality.

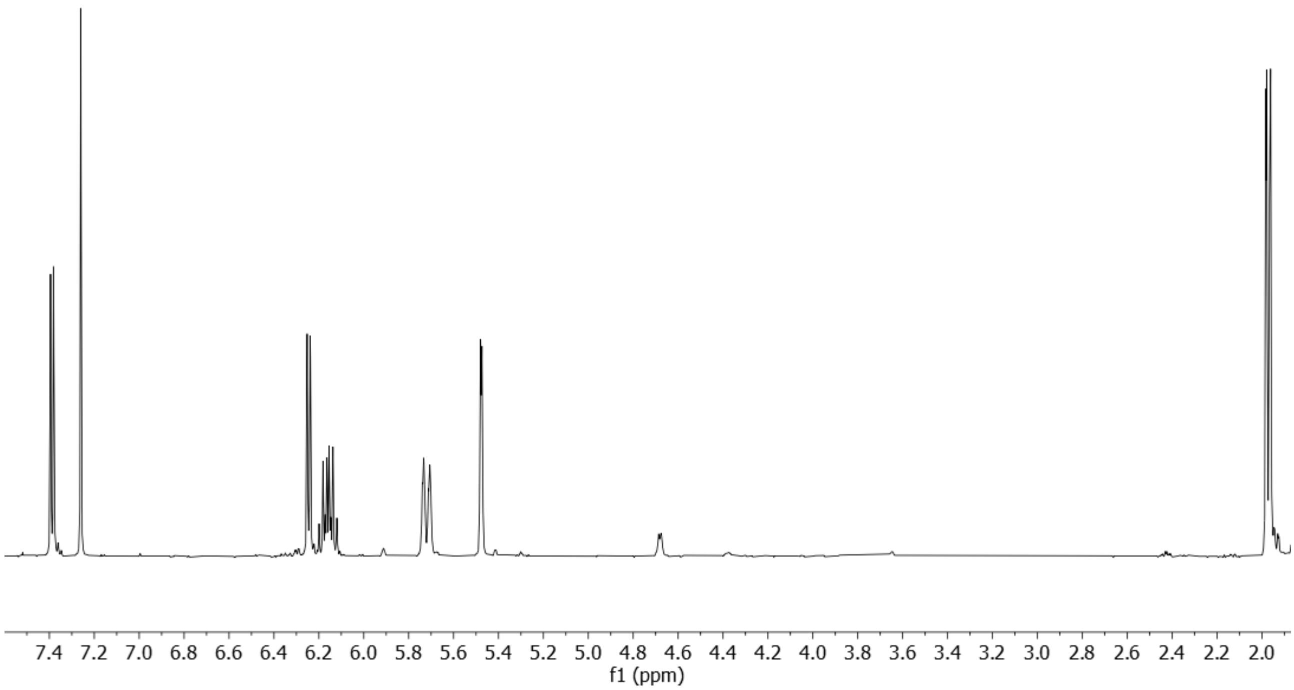

Figure S5. ^{1}H-NMR spectrum of (4*Z*,8*Z*)-matricaria lactone (**3**) recorded in $CDCl_3$ at 500 MHz.

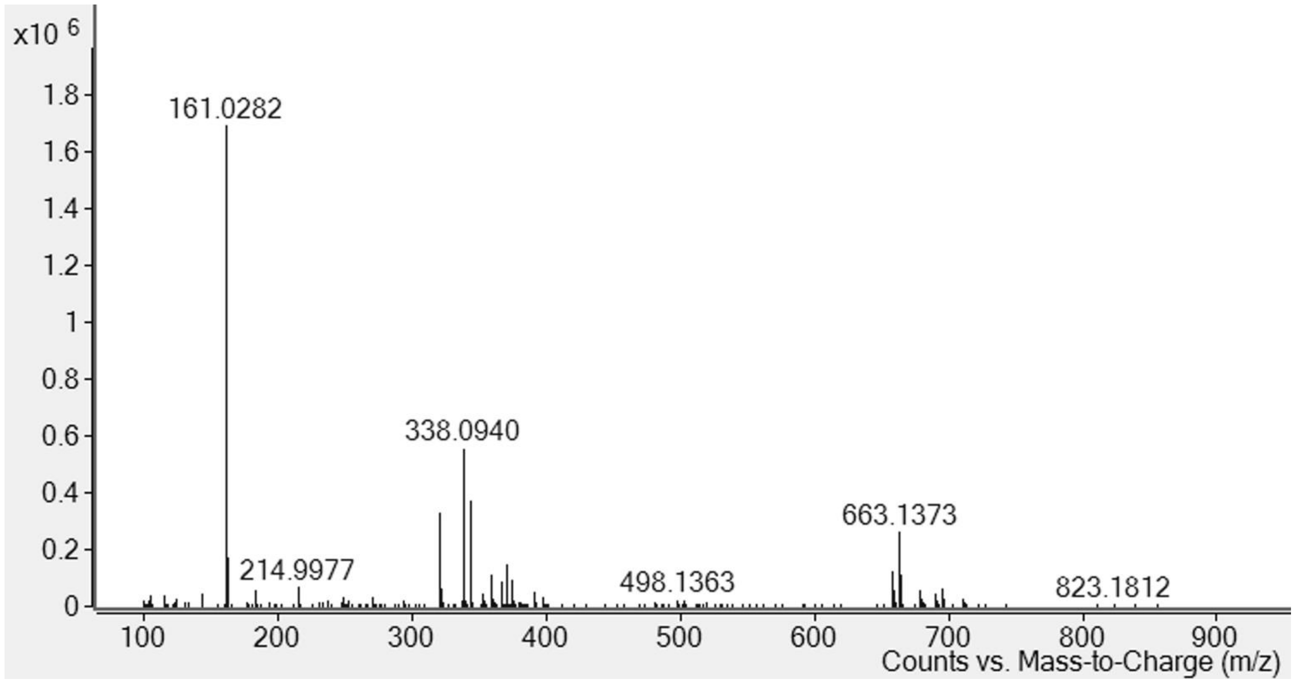

Figure S6. ESI MS spectrum of (4*Z*,8*Z*)-matricaria lactone (**3**) recorded in positive modality.

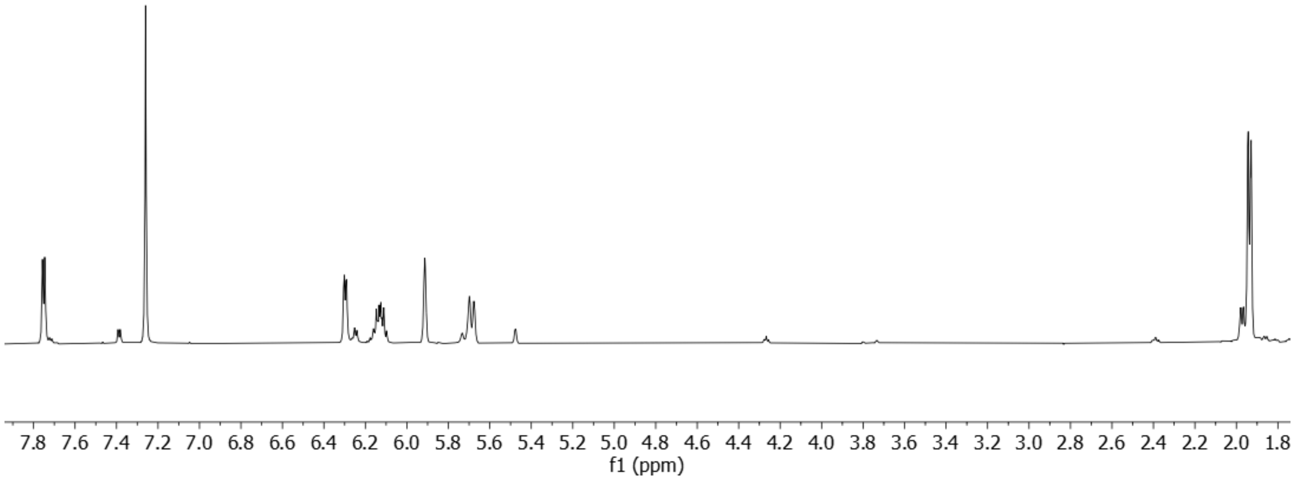

Figure S7. ^{1}H-NMR spectrum of (4*E*,8*Z*)-matricaria lactone (**4**) recorded in $CDCl_3$ at 500 MHz.

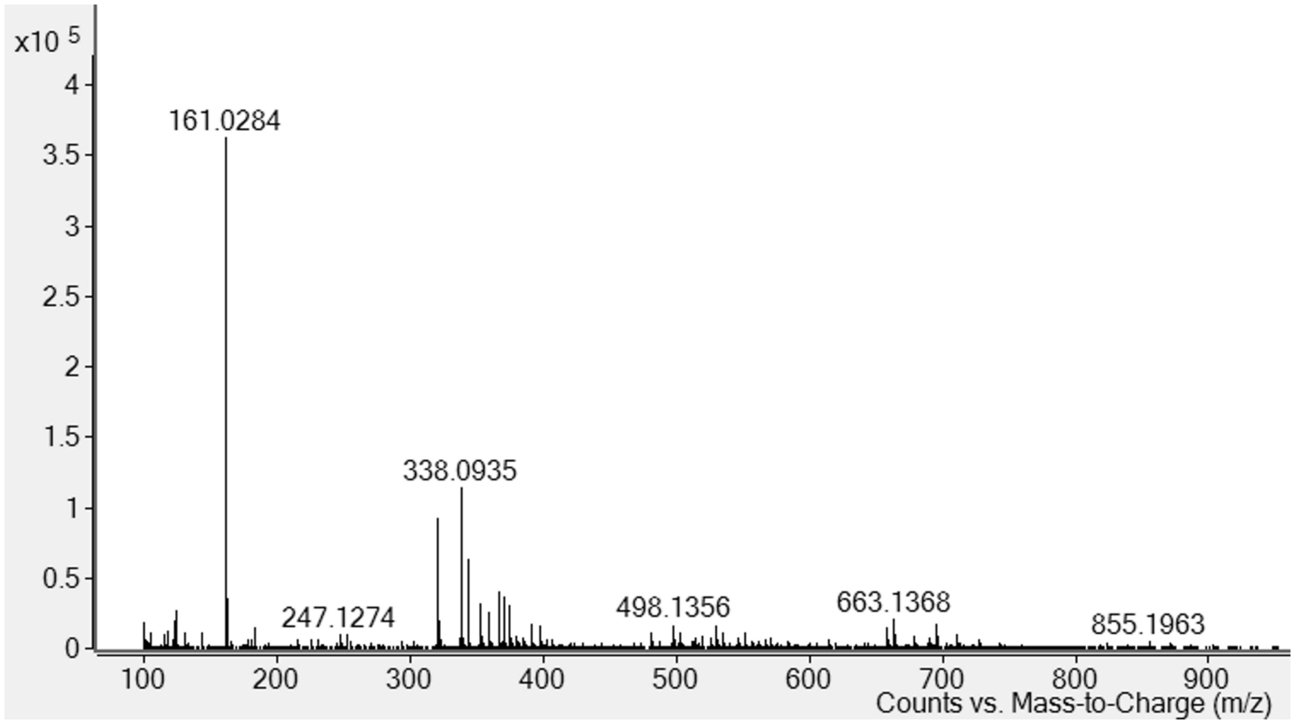

Figure 8. ESI MS spectrum of (4*E*, 8*Z*)-matricaria lactone (**4**) recorded in positive modality.

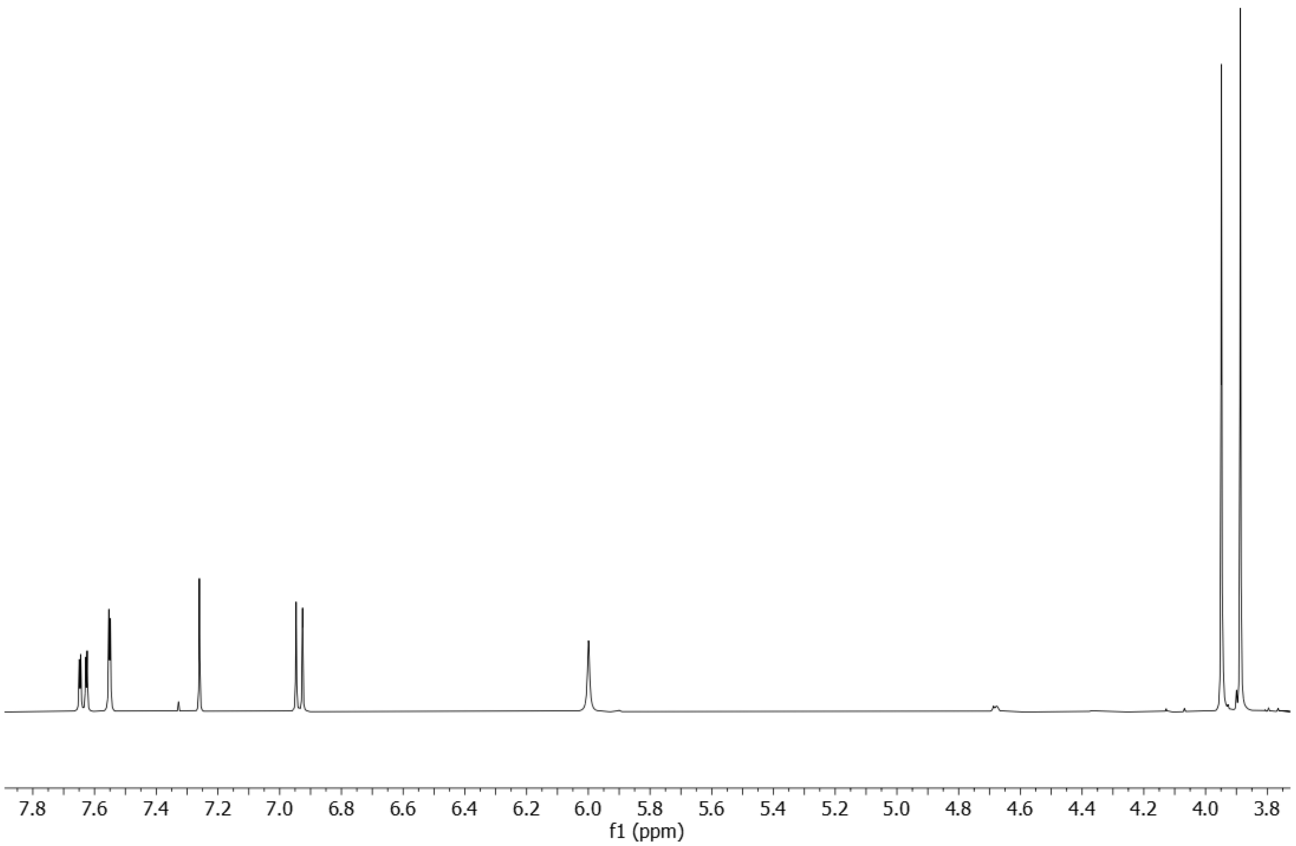

Figure S9. ^{1}H-NMR spectrum of methyl 4-hydroxy-3-methoxybenzoate (**5**) recorded in $CDCl_3$ at 500 MHz.

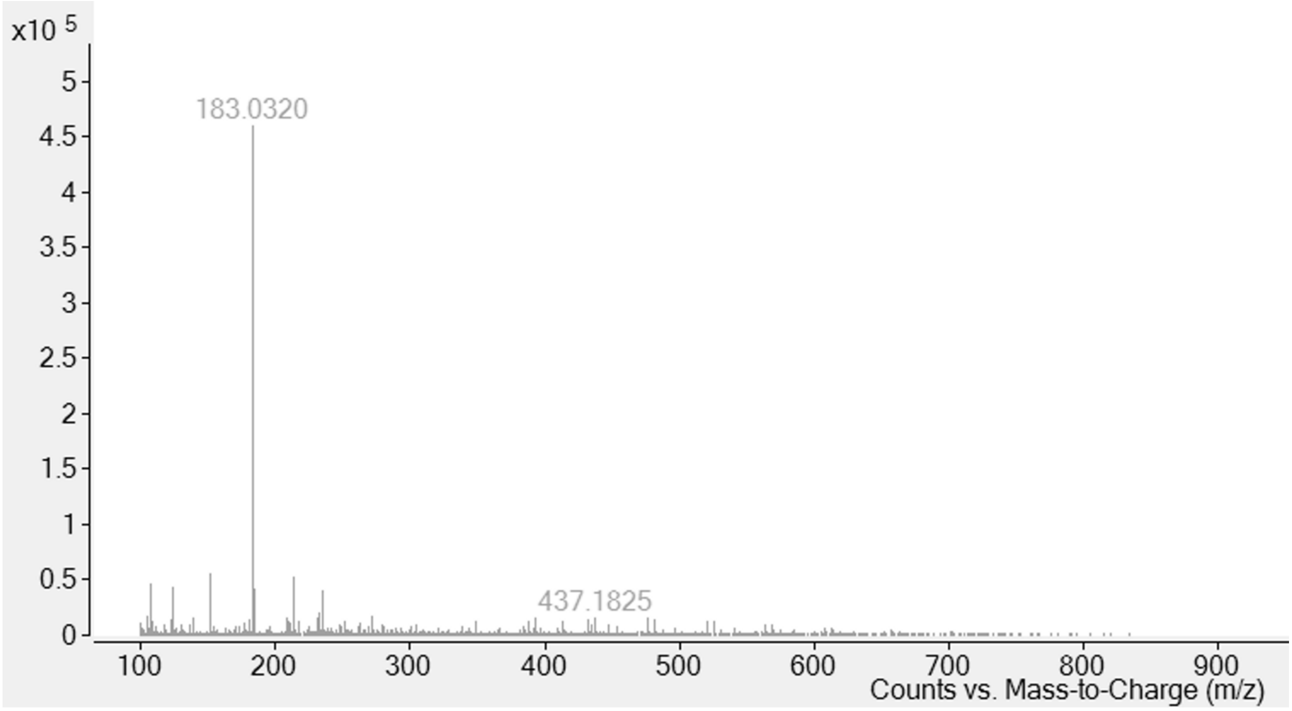

Figure S10. ESI MS spectrum of methyl 4-hydroxy-3-methoxybenzoate (**5**) recorded in positive modality.

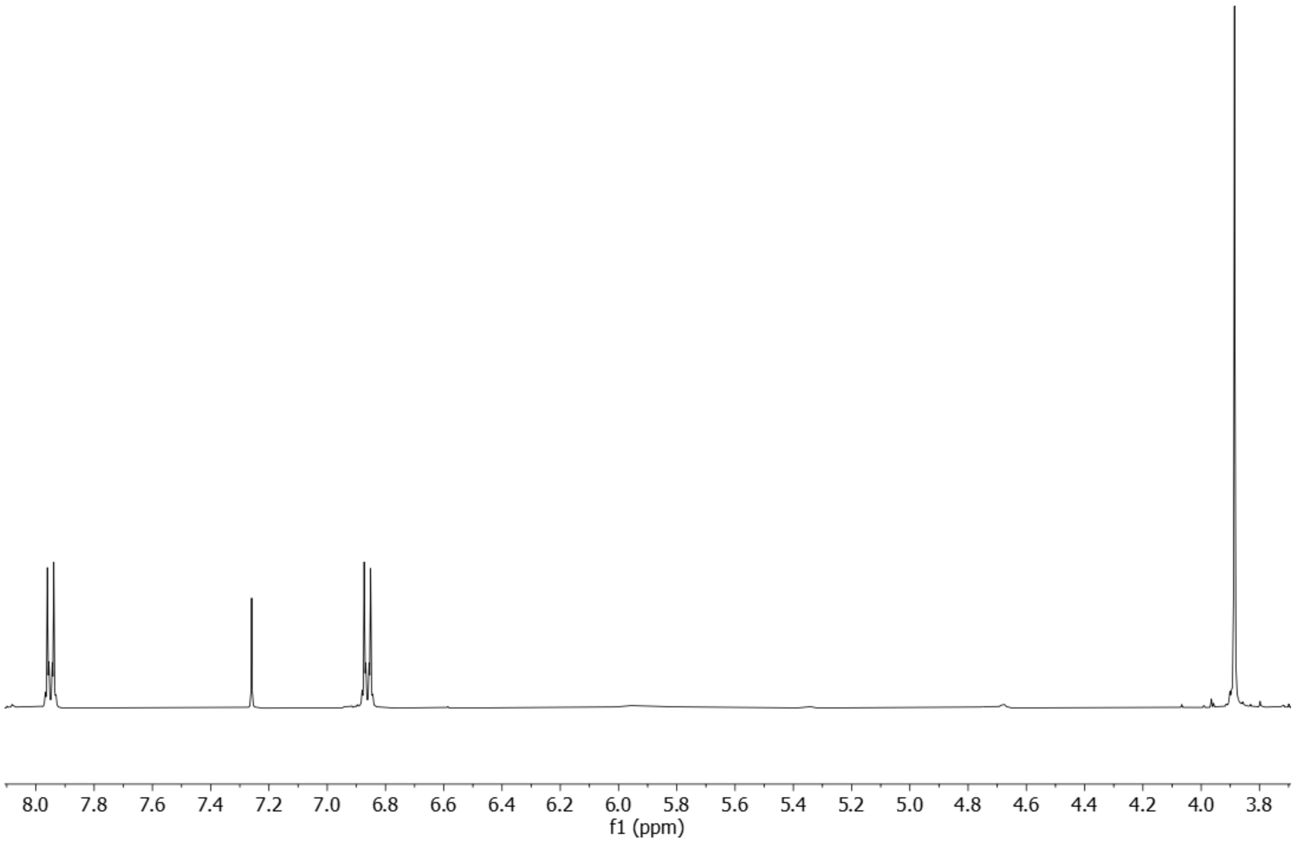

Figure S11. ^{1}H-NMR spectrum of methyl 4-hydroxybenzoate (**6**) recorded in $CDCl_3$ at 500 MHz.

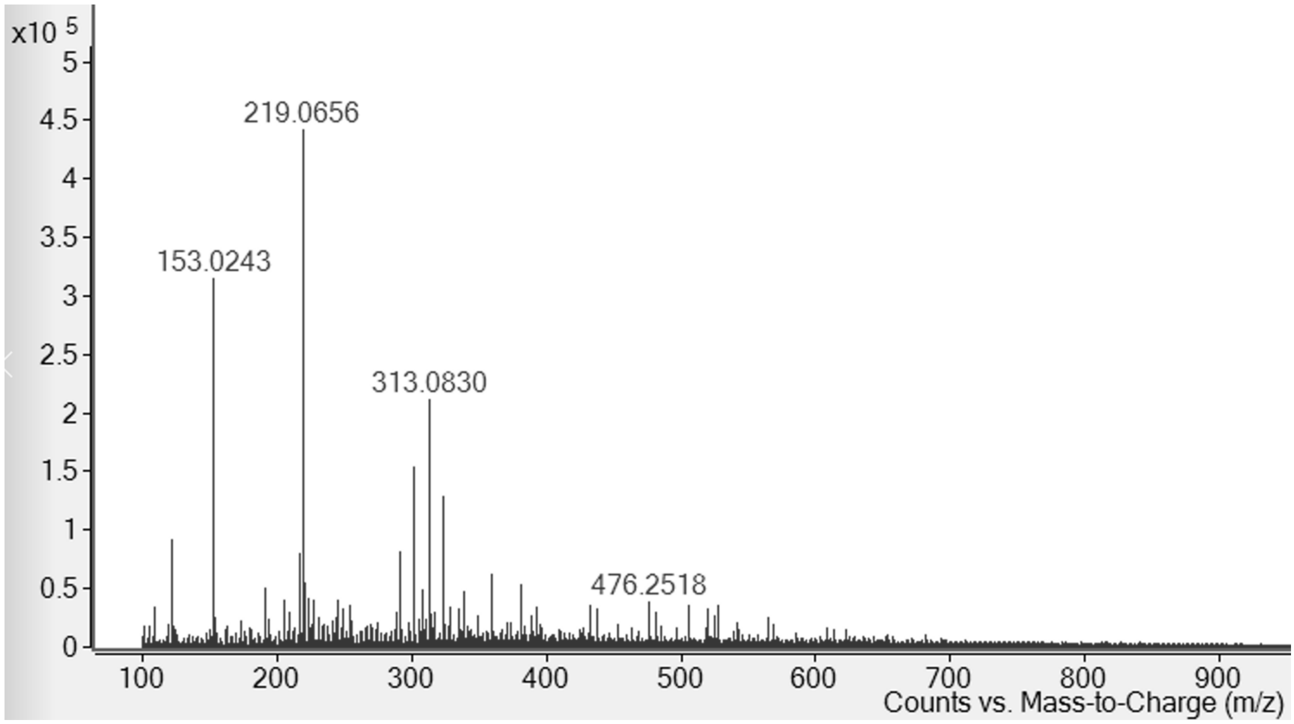

Figure S12. ESI MS spectrum of methyl 4-hydroxybenzoate (**6**) recorded in positive modality.

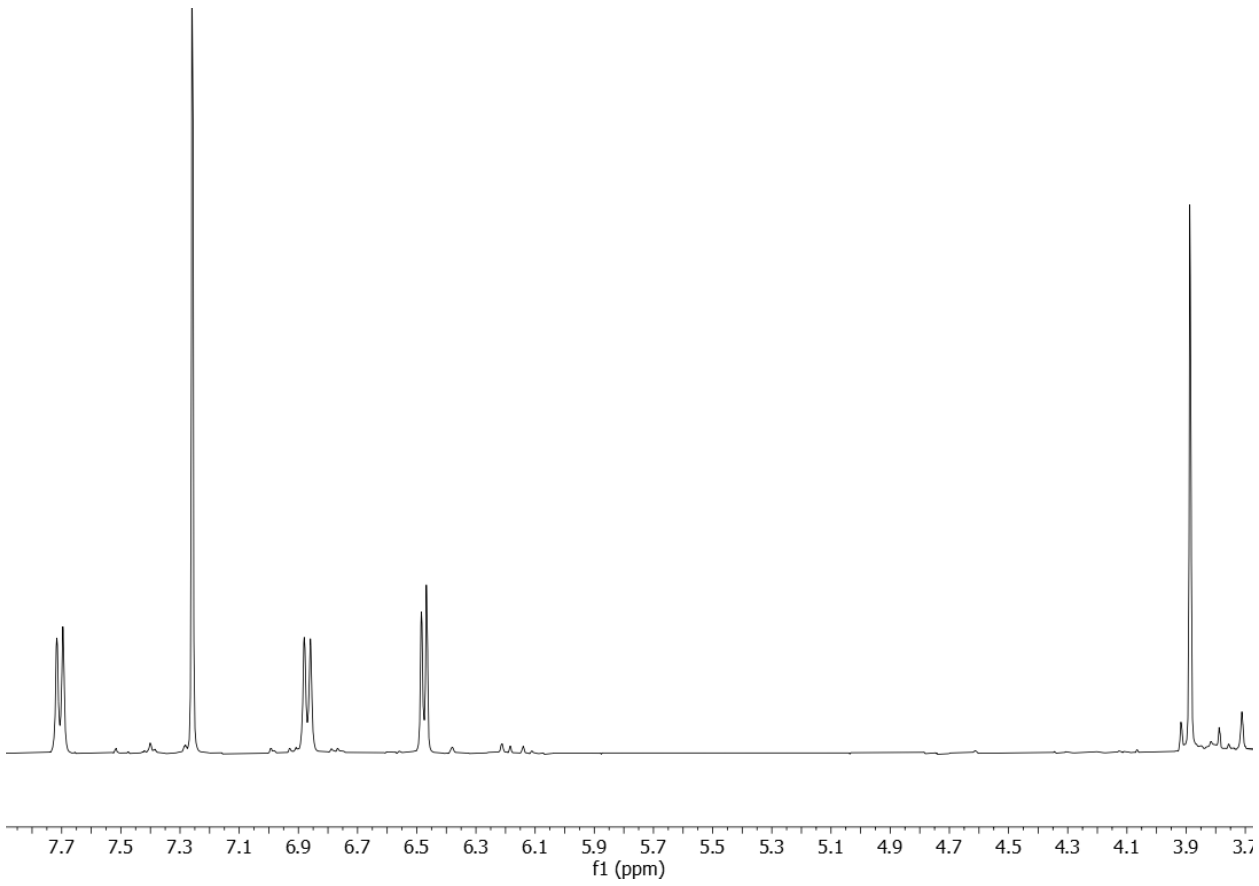

Figure S13. ^{1}H-NMR spectrum of hispidulin (**7**) recorded in $CDCl_3$ at 500 MHz.

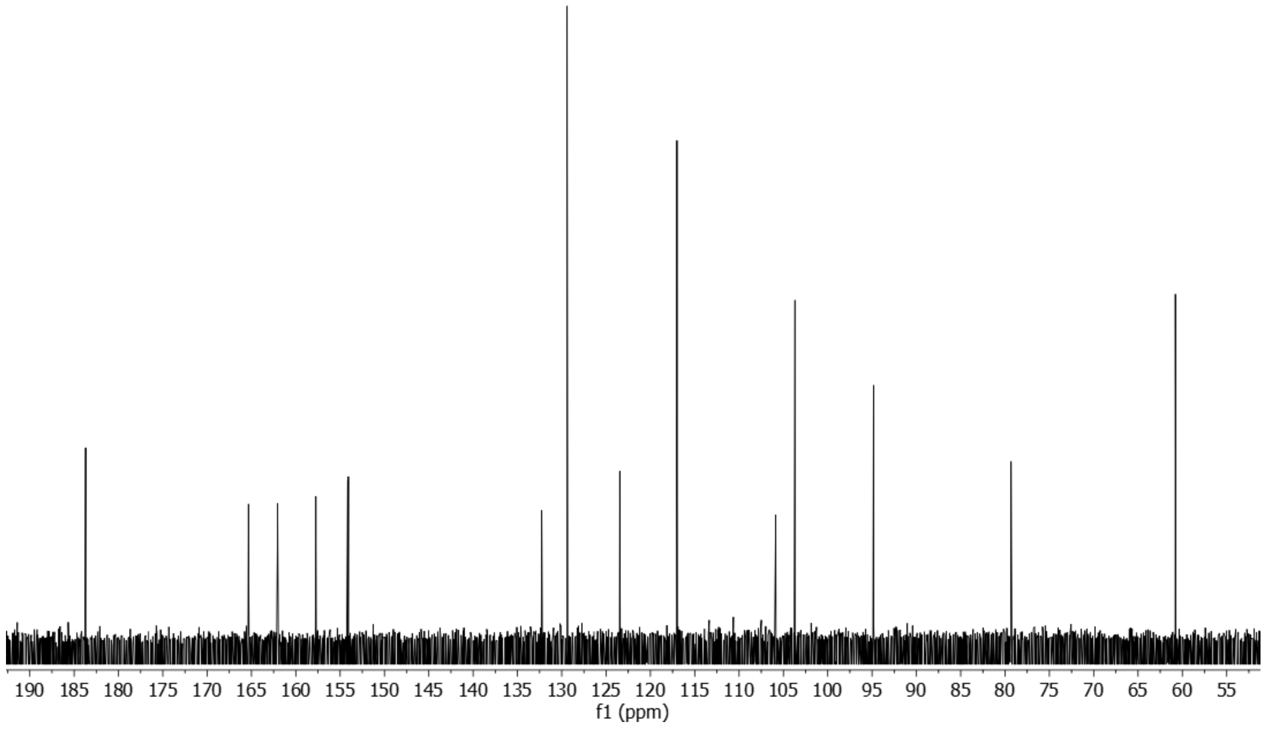

Figure S14. ^{13}C-NMR spectrum of hispidulin (**7**) recorded in $(CD_3)_2CO$ at 125 MHz.

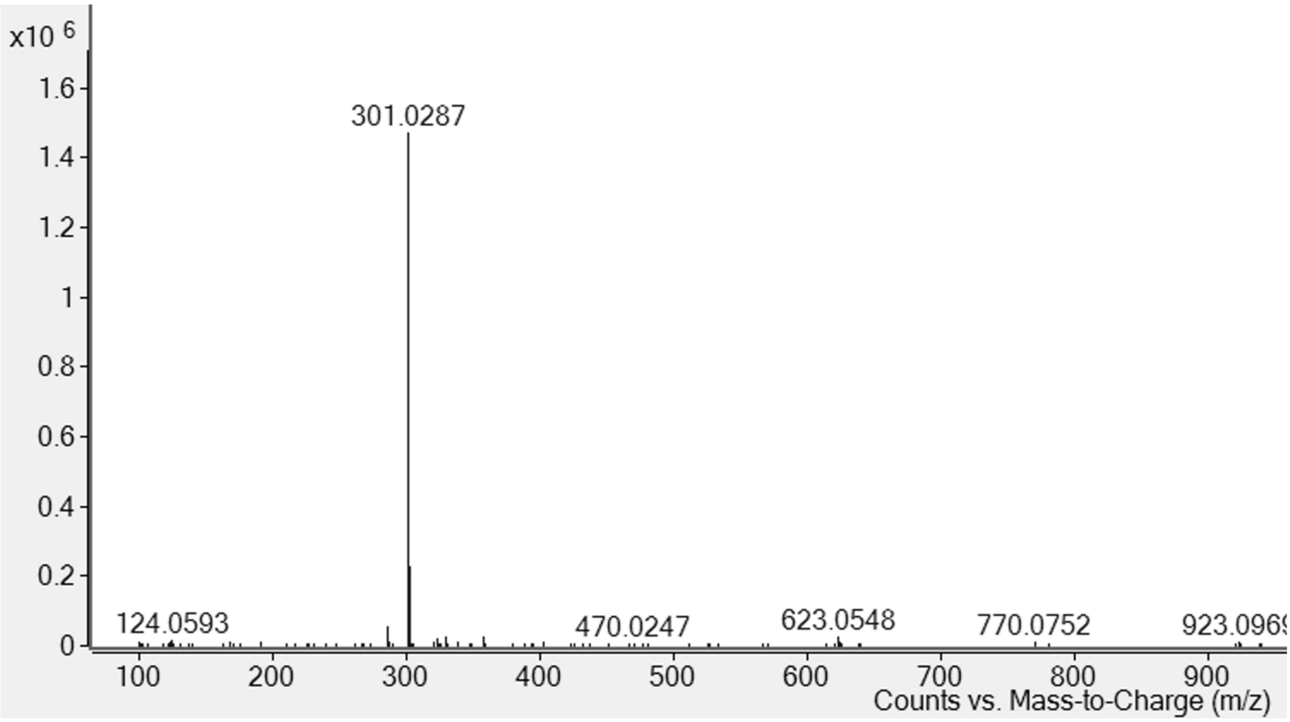

Figure S15. ESI MS spectrum of hispidulin (**7**) recorded in positive modality.
